# Central nervous system stimulants for secondary attention deficit-hyperactivity disorder after paediatric traumatic brain injury: a rationale and protocol for single patient (n-of-1) multiple cross-over trials

**DOI:** 10.1186/1471-2431-13-89

**Published:** 2013-05-28

**Authors:** Hugh EJ Senior, Lynne McKinlay, Jane Nikles, Philip J Schluter, Sue-Ann Carmont, Mary-Clare Waugh, Adrienne Epps, Owen Lloyd, Geoffrey K Mitchell

**Affiliations:** 1 Discipline of General Practice, School of Medicine, The University of Queensland, 11 Salisbury Rd, Ipswich 4305, Australia; 2 Queensland Children’s Medical Research Institute, Level 4, Foundation Building, Royal Children's Hospital, Herston, Queensland 4029, Australia; 3 School of Health Sciences, University of Canterbury, 20 Kirkwood Ave, Ilam 8041, New Zealand; 4 School of Nursing and Midwifery, The University of Queensland, Queensland 4305, Australia; 5 Rehabilitation Department, The Children's Hospital at Westmead, Cnr Hawkesbury Rd and Hainsworth St, Westmead, Sydney, NSW, 2145, Australia; 6 Rehabilitation Department, Sydney Children's Hospital, High St, Randwick, NSW 2031, Australia

**Keywords:** Traumatic brain injury, TBI, Children, Methylphenidate hydrochloride, MPH, Dexamphetamine, n-of-1 trial

## Abstract

**Background:**

It is estimated that 22,800 children were living with an Acquired Brain Injury (ABI) (0.6% of children aged under 15 years) in Australia during 2003. Many children after a traumatic brain injury will experience difficulties with attention and concentration; a condition termed secondary Attention Deficit-Hyperactivity Disorder. There is conflicting evidence on whether treatment with stimulant therapy with medications such as methylphenidate or dexamphetamine will improve the attention and behavior of children with this condition.

**Methods/Design:**

Single patient trials (n-of-1s or SPTs) evaluate the effect of titrated doses of psychostimulants methylphenidate or dexamphetamine compared to placebo on attention and behavior, in children with TBI and secondary ADHD. The aggregation of multiple SPTs will produce a population estimate of the benefit. Forty-two children will be registered into the trial through rehabilitation services at three large children’s hospitals in Australia. Patients will complete up to 3 cycles of treatment. Each cycle is 2 weeks long comprising seven days each of treatment and placebo, with the first two days of each cycle considered a washout period and the data not analysed. The order of treatment and placebo is randomly allocated for each cycle. The Conners’ Parent Rating Scales long forms will be employed to measure change in attention-deficit/hyperactivity and related problems of the child, and the primary outcome measure is the Conners’ Global Index Parent Version. Secondary outcomes include the teacher and child (if aged > 12 years) Conners’ Rating Scales, the Behaviour Rating Inventory of Executive Function among other measures. This study will provide high-level evidence using a novel methodological approach to inform clinicians about the most appropriate treatment for individual children. Through aggregation of individual trials, a population estimate of treatment effect will be provided to guide clinical practice in the treatment of children with secondary ADHD after a traumatic brain injury.

**Discussion:**

This study employs an innovative methodological approach on the effectiveness of CNS stimulants for secondary ADHD from a brain injury. The findings will both guide clinicians on treatment recommendations, and inform the concept and acceptance of SPTs in paediatric research.

**Trial registration:**

Australian New Zealand Clinical Trials Registry. ACTRN12609000873224

## Background

During 2003, one in forty-five (432,700) people in Australia experienced an acquired brain injury (ABI) causing a disability with activity limitations or participation restrictions [1]. It is estimated that 22,800 children were living with an ABI (0.6% of children aged under 15 years) in Australia during 2003 [2].

Consequences for children with TBI are difficulties with attention, concentration and self-regulation [3,4]. Up to 4 years after TBI, a fifth to one-half of the children will have a clinically significant attention disorder [5]. TBI derived attention disorders are comparable to developmental forms of Attention Deficit Hyperactivity Disorder (primary ADHD) and have been termed ‘Secondary ADHD’ [6]. Symptoms of secondary ADHD include clinically significant difficulties with attention, concentration, impulse control and hyperactivity, although TBI associated hyperactivity may be less severe than with primary ADHD [6].

Central Nervous System Stimulants are often a first-line treatment option in the treatment of primary ADHD [7], but there is a scarcity of well-designed studies on the efficacy of stimulant therapy for reducing the attention, concentration and impulsivity symptoms of secondary ADHD in paediatric populations. One double-blind, placebo-controlled, cross-over trial evaluated the efficacy of methylphenidate hydrochloride (MPH) (up to 10 mg per day over four days) on attention, memory, behaviour, processing speed and psychomotor skills of children (n=10, aged 5–16 years) who had experienced a TBI. The injury ranged from mild to severe and participants were clinically consistent with an ADHD diagnosis. No significant change occurred in any of the measures [8]. In contrast, Mahalick et al. describe a double-blind, placebo-controlled cross-over trial in children (n=14, aged 5–14 years) with a range of head injury severity who had acquired attentional disorders secondary to injury, evaluating MPH at a dose of 0.3 mg/kg twice daily over 14 days. A significant treatment effect was found on attention and concentration (p<0.05) [9]. Given the contradictory findings in the literature, and the potential benefits to children if effective, this protocol will describe a study that identifies both the children who will benefit and those who will not on a case-by-case basis and provides a population estimate of treatment effect using an aggregated n-of-1 trial design.

### N-of-1 trials

N-of-1 trials are multiple-cycle, double blind, placebo-controlled crossover trials using standardized measures of effect. The randomisation order is independently generated for each patient and they provide the strongest evidence about treatment efficacy in an individual patient [10]. This may be particularly important in the population of children with ABI, as some individuals are more likely to respond than others [9,11]. At trial’s end for the individual, the randomisation order is revealed and the treatment effect for the specific individual is determined based on response while on treatment versus control. By aggregating the results of all of the n-of-1 trials testing a particular drug, a treatment effect can be established for the patient population [12-14].

## Methods/Design

### Study aims

This study will test the efficacy of stimulant therapy with MPH or dexamphetamine (DEX) to improve attention and concentration, and executive dysfunction (including disorders of behavioural and emotional regulation) in children with traumatic brain injury compared to placebo by providing both a population and an individual estimate of treatment effect. A secondary aim is to evaluate the feasibility of n-of-1 trials as a means of conducting clinical trials within paediatric rehabilitation.

### Study design

**Figure 1:**
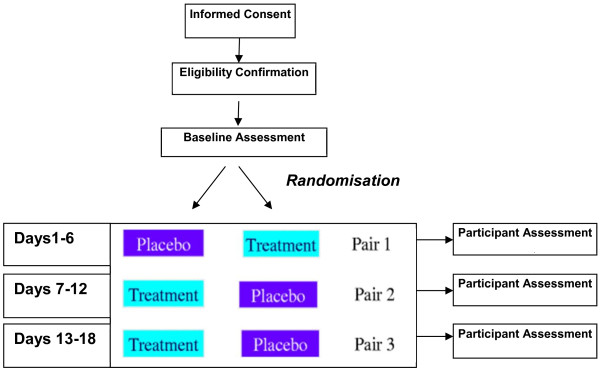
**Example of n**-**of**-**1 Design Schema**^**1**^**.**

A series of randomised, double-blind, placebo-controlled, single patient (n-of-1) trials conducted in three centres. Each participant will undergo three cycles, each of 2 treatment periods. The duration of each treatment period will be 1 week, making a total of 6 weeks per participant to complete the full trial. The first 2 days of each treatment period will not be used to assess efficacy, to allow for washout of active medications (half-lives: MPH 4 hours; DEX 6–8 hours). In each cycle, the drugs (stimulant or placebo) are randomly allocated to the participating child with both investigators and the participant blinded (see Figure 1). At the end of the trial, the order of medications is unmasked, and compared with the parent and teachers’ observations of the child’s behaviour. Repeated outcomes in the same direction favouring the treatment are reported in terms of a probability that the finding is correct. Multiple n-of-1 trials will be aggregated to produce a population estimate of the effect of stimulants.

### Study population and recruitment

Children who are outpatients will be recruited from three participating sites, the Brain Injury Service at the children's hospital at Westmead, the Queensland Paediatric Rehabilitation Service and Sydney Children’s Hospital. Participants will be invited to participate if they are aged between 6 and 16 years old, have a clinical diagnosis of moderate to severe brain injury, and are at least 12 months post-injury. Severity of brain injury is based on duration of loss of consciousness, initial Glasgow Coma Scale Score (GCS) at presentation to the treating hospital and duration of post-traumatic amnesia (PTA). Moderate TBI is defined as a loss of consciousness for 30 to 60 minutes, or GCS 9–12, or PTA from 1 day to 1 week; and severe TBI as loss of consciousness > 60 minutes, or GCS < 9, or PTA > 1 week. They must also have a clinically diagnosed and significant attention/concentration disorder or executive dysfunction (including behavioural or emotional regulation disorders) that may respond to stimulants. The participant must have at least two people (parent or other person, teacher) who can consistently monitor the child’s symptoms to allow data collection. Children are excluded if they have any of the following clinical conditions: uncontrolled seizure disorder, moderate to severe hypertension, clinically significant anxiety, motor tics, Tourette syndrome, suspected or proven cardiac conduction problems, idiosyncratic reaction to sympathomimetic amines, or history of drug abuse (including high caffeine beverages and appetite suppressants). Other criteria for exclusion are if the parent is unable to fill out forms in English or the child’s school is unwilling to participate.

### Primary outcomes

The child (if aged > 12 years), parent and the child’s teacher will each complete a weekly diary containing the primary and secondary outcome measures. The Conners’ Parent Rating Scales long forms will be employed to measure change in attention-deficit/hyperactivity and related problems of the child and the primary outcome measure is the Conners’ Global Index Parent Version (CGI-P) [15]. Conners’ is a widely used measure of attention-deficit/hyperactivity and related problems for children aged 8 to 18 years. Subscales include inattention, hyperactivity/impulsivity, learning, executive functioning, aggression and peer relations, as well as subscales mapping onto DSM-IV criteria [16] for ADHD (inattentive), ADHD (Hyperactive-impulsive), ADHD combined, Conduct Disorder, and Oppositional-Defiant Disorder. The CGI-P is a brief measure of general psychopathology comprised of 10 items embedded within the long form rating scales. It is a measure of the severity of ADHD symptoms including hyperactivity and attentional deficits. Ratings are on a 4-point Likert scale, and are converted into normalised scores, if normative data is available.

### Secondary outcomes

Secondary measures include the teacher and child (if aged older than 12 years) Conners’ 3 long form rating scales for teacher or child [15]. Other secondary measures include the Behaviour Rating Inventory of Executive Function (BRIEF) questionnaire to be completed each by the parent and teacher [17]. This is a measure of executive functioning in the home and school environments. Additional data include diagnosing the presence or absence of ADHD using DSM-IV criteria [16], demographics, type and duration of symptoms due to brain injury, date of injury, and history of previous stimulant therapy.

### Randomisation

The trial is composed of 3 cycles, each with 2 treatment periods of a week, giving a total duration of 6 weeks. A computer generated randomisation schedule created by the study statistician and held by the site pharmacies (and not accessible to investigators) will predetermine the order of medication (stimulant (MPH or DEX) or placebo) in each cycle.

### Safety reviews

At each contact with the researcher and via the parent diary, adverse events will be recorded. Parents will be contacted weekly (or more frequently if there are any concerns) to ensure diary completion and to ask about any adverse events.

Seriousness, causality, severity and expectedness of adverse events will be evaluated. Cases that are considered serious, possibly, probably or definitely related to the drug and are unexpected are to be unblinded. Any study-related problem posing risk of harm to participants and any type of serious adverse event will be reported to the institutional ethics committee and to the Data and Safety Monitoring Board (DSMB).

### Treatment, concomitant medications and compliance

The study drug to be tested is Methylphenidate or Dexamphetamine versus Placebo. The dose to be trialled will be individually titrated to each child as per accepted clinical practice, prior to randomisation (see below).

Participants will be randomly assigned to each of the following medications in each of the three cycles.

1. Active medicine

a. MPH - maximum dose 0.3 mg/kg

a. MPH – long acting tablets (on pre-trial dose) OR

a. DEX - maximum dose 0.15 mg/kg/DOSE

2. Placebo - visually matched capsule at the same dose as test medicine.

One 5 mg tablet dexamphetamine and one 10 mg tablet methylphenidate are equipotent. The pharmacist will purchase the trial medication and placebo, organize encapsulation, and package trial medications at the titrated dose in Webster packs according to the randomisation schedule. The pharmacy will produce 3 × 1 week’s supply of active drug and 3 × 1 week’s supply of placebo, in capsule form. Compliance will be determined by capsule count at the end of the study.

### Pre-trial dose titration of study medication

There is variation in required dose amongst children under treatment. If the child is not already stabilised on methylphenidate or dexamphetamine, prior to the trial commencement he/she will be stabilised on an appropriate individualised dose. They will be stable on this dose for approximately two weeks prior to commencement of the trial, unless already on stimulant medication prior to recruitment. The appropriate dose is one that provides a clinical improvement in target behaviours, and is well-tolerated with minimal side-effects, up to the maximal recommended dose. If unacceptable side effects occur while taking one of the stimulants, consent will be sought to enrol into the trial using the other stimulant. Children who were prescribed long acting methylphenidate before the trial, and the treating clinician’s assessment is they were likely to benefit from long-acting MPH, will be invited to participate in the trial with long-acting MPH as the trial test drug, on a case-by case basis. Others will be offered short-acting MPH or DEX.

### Post-trial treatment for individual patients

Once the analysis has been completed for a specific child, a report detailing the findings, adverse events during placebo and treatment, and recommendations to continue or stop treatment with trial medication is sent to the child’s paediatrician. An appointment is made for the child to discuss the results with their doctor and to make a decision regarding further treatment with MPH or DEX. Clinicians who recruit participants will most likely also be responsible for receiving the post-treatment report. This potentially may unblind the clinician about the possible effectiveness of the test drug after viewing a number of post-treatment reports. However, any selection bias is minimised in the study design as all participants randomly receive both the treatment and placebo therapies. Observer bias is minimised as data is collected from the parent, teacher (and child) independent of the clinician.

### Statistical considerations

#### Sample size and feasibility

The Conners’ Parent Rating Scales (long form) will be employed to measure change in attention-deficit/hyperactivity and related problems of the child with the total CGI-P score as the primary endpoint in this study to calculate the sample size. Currently, there are no validated scales that measure hyperactivity in children with acquired brain injury; the Conners’ Scales are designed for children without brain injury, and are used in ADHD diagnosis. As normative scales for the Conners’ Rating Scale have yet to be created for the study population, a pilot study employing the same methodology (n=10) was undertaken to ascertain completion rates and parameter estimates. In this pilot, 80% completed cycle I, 60% completed cycles I and II, 50% completed cycles I, II and III, the mean treatment difference equalled 2.4 (SD 6.2), and the within-patient serial correlation between repeated measures was 0.24. Applying these completion rates and estimates, assuming no period effect or treatment×time interaction, the study statistician (PS) designed a computer-based simulation model in Stata version 12.0 statistical software (StataCorp, College Station, Tex, USA) to derive the required sample size. Using simulations of size N=10,000, and defining statistical significance as α=5% (2-sided), 70 patients need to be enrolled to detect a mean treatment mean difference in the total CGI-P score of 2.0 with 80% power. Of the 70 patients, it is expected that 35 patients will complete cycles I, II and III, 7 patients will complete cycles I and I, 14 patients will complete cycle I, , and 14 patients are not expected to complete any cycles. In a similar single cycle trial of stimulants in ABI, Bakker and Waugh [18] recruited 25 participants from 3 sites over one year, with 1500 children (aged 6 to 12 years) on the combined brain injury database of 2 of these sites. We estimate it is feasible to recruit 70 participants over 3 sites as we will recruit children over a wider age range and a recruitment period of two years.

#### Statistical analysis

Measures on the Rating Scales will be compared within pairs of treatment periods for each trial. We will calculate adjusted mean differences in attention, hyperactivity (measured on the Conners’ instrument) and other variables such as aggression, impulsivity, risk-taking behaviour, fatigue scores between treatments using hierarchical Bayesian (HB) random effects models. For details of the advantages of Bayesian approaches over conventional frequentist statistical methods, refer to Zucker et al. 1997, Schluter and Ware 2005, and Nikles et al. 2011 [11-13]. In brief, the Bayesian approach allows both individual and aggregate analyses to be simultaneously conducted even when the number of completed cycles varies between patients. The method incorporates natural hierarchies and serial correlations (e.g. clustering by physician, setting or location), the outcome variables can take any parametric functional form, and relevant information sourced from within the trial and elsewhere can be included to produce coherent estimates and confidence intervals [11-13]. Frequentist methods offer no such guarantee [12,13]. Despite these advantages, Bayesian approaches are not routinely employed in medical research as they are often perceived as being analytically complex and difficult to conduct. However, a cost free and easily implementable downloadable software programme, WinBUGS, [19] challenges this perception and will be used in this study (see: http://www.mrc-bsu.cam.ac.uk/bugs/winbugs/contents.shtml).

We will assume a clinically important difference of 0.3 standard deviations for all scores. For normally distributed data, we will follow the method advocated by Zucker and colleagues, [13] and use their standard non-informative prior and hyperprior distributions. For binary data, we will employ the corresponding non-informative prior specifications and methods described by Schluter and Ware [12]. The likelihood distributions will be assessed for violations and data transformations undertaken, where necessary. Also, as we are conducting the study over multiple sites, an apposite hierarchy structure will be introduced into all models that accommodates this clustering [13]. To describe participants’ overall response, three types of Bayesian results will be presented: (i) the mean of the posterior distribution of the mean difference between placebo and stimulant scores, which gives the best estimate of the overall effect size difference between treatments; (ii) the associated 95% credible region, which give intervals of uncertainty (in this case the 2.5 and 97.5 percentile) of the posterior distributions used in (i); and (iii) the posterior probability of the mean difference that stimulant scores were better than placebo scores, which describes the likelihood that the patients will favour the active treatment in future cycles [13]. A patient will be defined to be a ‘responder’ when these estimated values exceed predefined threshold values [12].

## Ethical considerations

### Ethical approval

Written approvals have been obtained from relevant Hospital Research Ethics Committees (Royal Children’s Hospital and Health Service District Ethics Committee, Queensland Health; The Children’s Hospital at Westmead HREC, Sydney) and The University of Queensland Medical Research Ethics Committee prior to study commencement. Approval was obtained from Education Regulatory Authorities in Queensland and NSW (Queensland Government Dept. of Education and Training, Catholic Education Archdiocese of Brisbane, NSW Government of Education and Communities, Catholic Education Office Sydney). Informed consent is required from the parent and teacher (and assent for children aged >12 years). All patients receive a re-identifiable registration number for the trial CRFs and database.

### Trial withdrawal and discontinuation of trial medication

Patients can withdraw from the trial at any time without reason or impact on usual care. Reason for withdrawal is recorded. Only data from completed cycles will be included in the analysis. The trial will be stopped if DSMB recommend stoppage due to safety concerns.

## Discussion

In Australia, 0.6% of children aged under 15 years are estimated to have experienced an ABI [2], with a fifth to one-half of the children will have a clinically significant attention disorder at up to 4 years after injury [5]. This protocol describes aggregated N-of-1 trials to assess the efficacy of psychostimulants on attention, hyperactivity and high cerebral functions of children who have experienced traumatic brain injury. Current evidence from cross-over trials testing efficacy of psychostimulants in this population is inconclusive. Hence, additional high-quality evidence is required prior to recommending treatment.

Recruiting sufficient numbers of children who have experienced a TBI into traditional RCTs is problematic; therefore we propose an alternative methodology while providing high-level evidence. Due to their cross-over design, aggregated SPTs have a smaller required sample size than their conventional parallel arm RCT counterparts for equivalent levels of statistical power, and are better at controlling for confounding [20]. As every participant receives both the active and placebo treatments, this makes participation more attractive than in a conventional RCT where there is a chance of being randomised to the placebo arm of the trial. Finally, because the same person contributes multiple data points to both the active and placebo arms of the trial, the sample is perfectly matched.

An additional strength of this proposed study is the pre-trial titration of psychostimulants to ensure a dose was selected that appeared to produce positive behavioural result, while being well tolerated, to allow comparison with placebo for each individual child. For each child participant, at trial end a report on efficacy and adverse effects is provided to the parents and the child’s paediatrician. This is not possible in a traditional RCT. This study will be the first to use aggregated n-of-1 trials in a paediatric population with TBI. An important component of the study is not only to test the efficacy of MPH or DEX on cognitive and behavioural outcomes in children, but to evaluate the acceptability, methodology and analytical aspects of employing this methodology in this patient population. This information will contribute to proof-of-concept and ensure the acceptance of this method as a valuable and reliable research tool especially in drug trials where populations are difficult to recruit. As no previous n-of-1 trials have been conducted in this area, this reported series of trials significantly enhances paediatric community rehabilitation knowledge and practice.

## Abbreviations

ABI: Acquired brain injury; ADHD: Attention deficit-hyperactivity disorder; BRIEF: Behaviour rating inventory of executive function; CGI-P: Conners’ global index- parent version; DSMB: Data safety monitoring committee; DEX: Dexamphetamine; ECBI: Eyberg child behaviour inventory; GCS: Glasgow coma scale score; MPH: Methylphenidate; N-of-1: Single participant trials; PTA: Post-traumatic amnesia; RCT: Randomised controlled trial; SPT: Single participant trials; SUSAR: Suspected unexpected serious adverse reaction; TBI: Traumatic brain injury

## Competing interests

The authors declare that they have no competing interests to declare.

## Authors’ contributions

LM, GM, JN, PS conceived the study. HS was responsible for drafting the manuscript. All authors contributed to the design of the study. All authors contributed to and approved the manuscript.

## Pre-publication history

The pre-publication history for this paper can be accessed here:

http://www.biomedcentral.com/1471-2431/13/89/prepub
